# Probiotics’ efficacy in paediatric diseases: which is the evidence? A critical review on behalf of the Italian Society of Pediatrics

**DOI:** 10.1186/s13052-020-00862-z

**Published:** 2020-07-25

**Authors:** Massimo Martinelli, Giuseppe Banderali, Marisa Bobbio, Elisa Civardi, Alberto Chiara, Sofia D’Elios, Andrea Lo Vecchio, Mattia Olivero, Diego Peroni, Claudio Romano, Mauro Stronati, Renato Turra, Irene Viola, Annamaria Staiano, Alberto Villani

**Affiliations:** 1 Department of Translational Medical Science, Section of Pediatrics, University of Naples “Federico II”, Via S. Pansini, 5, 80131 Naples, Italy; 2 Clinical Department of Pediatrics and Neonatology, San Paolo Hospital, ASST Santi Paolo e Carlo, University of Milan, Milan, Italy; 3 ASL TO4 Piemonte, Turin, Italy; 4 Neonatal Intensive Care Unit, Fondazione IRCCS Policlinico San Matteo Pavia, Pavia, Italy; 5 Department of Clinical and Experimental Medicine, Section of Pediatrics, University of Pisa, Pisa, Italy; 6 Pediatric Gastroenterology and Cystic Fibrosis Unit, University of Messina, Messina, Italy; 7 Pediatric and Infectious Disease Unit, Bambino Gesù Children’s Hospital, IRCCS, Rome, Italy

**Keywords:** Probiotics, Paediatrics, Necrotizing enterocolitis, Acute infectious diarrhoea, Allergy, Functional gastrointestinal disorders

## Abstract

During the last decade several paediatric studies have been published with different possible indications for probiotics, leading to a global increase of probiotics’ market. Nevertheless, different study designs, multiple single/combined strains and small sample size still leave many uncertainties regarding their efficacy. In addition, different regulatory and quality control issues make still very difficult the interpretation of the clinical data. The objective of this review is to critically summarise the current evidence on probiotics’ efficacy and safety on a different number of pathologies, including necrotizing enterocolitis, acute infectious diarrhoea, allergic diseases and functional gastrointestinal disorders in order to guide paediatric healthcare professionals on using evidence-based probiotics’ strains. To identify relevant data, literature searches were performed including Medline-PubMed, the Cochrane Library and EMBASE databases. Considering probiotics strain-specific effects, the main focus was on individual probiotic strains and not on probiotics in general.

## Introduction

The International Scientific Association for Probiotics and Prebiotics (ISAPP) has recently revised the definition of probiotics [1]. The selected international panel recognised that probiotics’ definition provided in 2001 WHO/FAO [2] was valuable and well-renowned among researchers, regulators and consumers and decided to only slightly rephrase it, as following: “live microorganisms that, when administered in adequate amounts, confer a health benefit on the host” [1]. In order to fulfil this definition, probiotics have to be present in a reasonable amount within the product. It has been suggested that at least 1 × 10^9^ colony-forming units (CFU) are needed in order to guarantee the passage through the gastrointestinal (GI) tract and the gut colonization for exerting a measurable beneficial effects [3]. A huge confusion regarding probiotics regulation still exists among different countries. In most cases probiotics have been recognised within the category “food supplements or dietary supplements”, while some regulatory agencies inquired whether they should be included within drugs’ umbrella, with all the consequences related to the more stringent pre and post-market safety criteria [4–6]. In Europe, probiotic-containing foods and food supplements are subjected to European Union regulation covered by the Food Products Directive and Regulation [7]. Since 2006, the European Food Safety Authority (EFSA) is responsible for food and food supplements evaluation. EFSA is consequently in charge for probiotics’ evaluation, in particular regarding health claims [8]. Accordingly to the European regulation, the Italian Ministry of Health confirmed the use of the word probiotics for food and food supplements under certain conditions, including a minimum number of viable cells (1 × 10^9^ CFU) administered per day, a full genetic characterization of the probiotic strain and a demonstrable history of safe use in the Italian market [9]. However, despite the existence of specific regulatory agencies, in 2017 the European Society of Pediatric Gastroenterology, Hepatology and Nutrition (ESPGHAN) Working group for Probiotics and Prebiotics released a position paper highlighting the need for improved quality controls [10]. In details, the authors after reviewing the literature, provided evidence of the inadequate quality of commercial probiotic products, in particular regarding microorganism specification, quantity, properties, and the presence of contaminants and concluded that more stringent quality control procedures are urgently needed and should be considered mandatory, also taking into account the vulnerability of paediatric population [10]. Differently from Europe, Food and Drug Administration already in 2007 released very stringent manufacturer instructions for food supplements, including probiotics [11]. Nevertheless, up to date these rules do not require control or verification of the effective quality of the product [11]. Despite the regulatory and quality control pitfalls, in the last decade a large number of paediatric studies, including randomized controlled trials (RCTs), and even systematic reviews and metanalyses have been published with different possible indications for probiotics, leading to a global increase of probiotics’ market [12–14]. The effects of probiotics have been highly strain-specific in relation to the different pathologies. Furthermore, a recently published paper by Zheng et al. clearly highlights how continuous changes of probiotics’ taxonomy may impair comparative analyses between different RCTs [15].

The aim of this review is to critically evaluate the literature on the efficacy and safety of specific probiotics strains in various paediatric diseases, including neonatal, allergic, infectious and GI disorders.

## Methods

A panel of experts was selected from across a range of paediatric disciplines within the Italian Society of Pediatrics. A face-to-face meeting resulted in organizing the manuscript into 4 different sections, choosing the most common topics for each different discipline, namely: *necrotizing enterocolitis and late onset sepsis*; *acute infectious diarrhoea (AID)*; *allergic diseases*; *functional gastrointestinal disorders (FGIDs)*. Each working subgroup was asked to perform a literature search using Medline-PubMed, the Cochrane Library and EMBASE databases with appropriate search strategies (available upon request) using a last search date of December 1st, 2019. Considering probiotics strain-specific effects, the main focus was on data on individual probiotic strains, rather than probiotics in general.

## Results

### Necrotizing Enterocolitis and late onset Sepsis

Necrotizing enterocolitis (NEC) and sepsis are leading causes of mortality and morbidity in premature infants; NEC has an incidence varying from 2,6 to 28% and an average mortality of 20–30%, up to 50% for infants requiring surgical treatment [16]. Late onset sepsis (LOS) occurs approximately in 20% of very low birth weight (VLBW) infants and has a significant overall mortality risk and potential long-term neurodevelopmental sequelae [17]. The onset of both conditions is rapidly progressive with involvement of multiple organs and treatment is unlikely to be wholly successful. Preventive strategies are urgently needed. The pathogenesis of NEC is multifactorial and incompletely understood, but the immaturity of intestinal barrier function and the development of an altered gut microbiota, with possible translocation of potentially pathogenic bacteria, may play an important role. Similar mechanisms are likely to be involved in cases of LOS when the infecting organism originates from a reservoir within the intestine [18–20]. The importance of a diverse intestinal microbiome for good health in later life has been long recognized. These bacteria have a range of roles, improving the intestinal immunological defences, preventing colonization and possible invasion by pathogens through the intestinal wall. After preterm birth, acquisition of the microbiome is slow, typically less diverse, and dominated by *Enterobacteriaceae* with relatively few *Lactobacilli* and *Bifidobacteria*. The contribution of abnormal patterns of the intestinal microbiota to the clinical onset of NEC and LOS is not clear, but there is evidence of perturbations of the gut microbiota in the period preceding the onset of clinical illness. The most recent Cochrane review of this topic, including 24 studies and more than 5500 infants, produced recommendations for the routine use of probiotics in prevention of NEC and mortality in preterm infants [21]. However, concerns about the efficacy and safety of probiotics in this population have limited their introduction into clinical practice. Actually, a recent survey demonstrated that probiotic provision varies between 0 and 100% in 153 different NICU in the world [22]. In the 5 years after the Cochrane review publication [21], more clinical trials and metanalyses have been performed to better evaluate the role of probiotics in the prevention of NEC and LOS in preterm infants. In 2015 Aceti et al. on behalf of the Italian Society of Neonatology, performed a new systematic review: the results show an overall benefit of probiotic supplementation for the prevention of NEC [23]. The 26 studies included in the metanalysis were extremely heterogeneous and in very few studies the same probiotic strain was used, weakening a strain specific sub-metanalysis. After pooling studies according to probiotic genus, no effect was documented for *Lactobacilli* and *Saccharomyces*; the analysis of studies using *Bifidobacteria* showed a significant efficacy of *Bifidobacterium breve (B. breve)* in reducing NEC [23]. Both these findings are in contrast with the Cochrane review [21]; the discrepancy may be due to differences in the studies included. Given the small number of trials reporting the rates of NEC in extremely low birth weight (ELBW) and intrauterine growth restriction (IUGR) infants, no specific recommendation was drawn in these two populations. In 2016, Costeloe et al. published a large RCT, the PiPs trial, recruiting more than 1000 babies [24]. The authors chose a single strain probiotic product (*B. breve BBG-001*) with the best available evidences at that time and an appropriate comparison group. No significantly differences were observed in LOS, NEC or death [24]. The disappointing results of the PiPs trial caused a major drawback for the use of probiotics in the management of the preterms. In 2017, Uberos conducted a retrospective cohort study evaluating clinical outcomes in VLBW infants, before and after the introduction of routinely probiotics supplementation with *Lactobacillus rhamnosus GG (LGG) or Lactobacillus acidophilus (L. acidophilus) and Lactobacillus bifidum (L. bifidum)* [25]. The authors observed a significant reduction in NEC ≥ Stage II (11.3 vs 4.8%), LOS (16 vs 10.5%) and mortality (19.4 vs 2.3%) in infants born before 32 weeks of gestational age (WGA), while in neonates aged ≤27 weeks the reduction was not statistically significant [25]. In the same year, Aceti et al., on behalf of the Italian Society of Neonatology published another metanalysis of 25 trials on probiotics and LOS: probiotics’ supplementation resulted in a significantly lower incidence of LOS (RR 0.79) [26]. According to feeding type, the beneficial effect was confirmed only in exclusively human milk (HM) fed preterm infants, probably because of a synergic action of probiotics and prebiotics compounds of HM (RR = 0.75) and only for probiotic mixtures [26]. Another review of 30 RCTs and 14 observational studies showed a reduction up to 50% of severe NEC and about 25% of all-cause mortality using of a two-probiotic combination *[L. acidophilus with Bifidobacterium infantis (B. infantis)]* [27]. Furthermore, a 12% reduction in the risk of sepsis in RCTs and a 19% reduction in observational studies were demonstrated. The metanalysis did not show a statistically significant effect in ELBW infants, because of insufficient number of clinical trials [27]. In 2018 the ESPGHAN Working Group on Probiotics, Prebiotics and Committee on Nutrition published a detailed strain specific and network metanalysis (NMA) of data on probiotics, used in preterm infants [28]. Differently from classical pair-wise meta-analyses which only address the comparative effectiveness among similar or competing interventions against a common comparator, NMA has the advantage to address multiple interventions simultaneously. Following this specific approach the authors were able to evaluate the efficacy of single probiotic strains or combination of studied strains [28]. Fifty-one trials and over 11000 newborns were included. Most of the different probiotic strains were evaluated in one or two trials, while only 5 were studied in at least 4 RCTs. Noteworthy, the review included moderately preterm infants because trials focused on smallest babies were limited. The meta-analysis shows that only a minority of probiotic strains has a statistically significant effect in reducing mortality and morbidity in preterm infants. The absence of significant effects may reflect a lack of adequately powered RCTs, or a genuine lack of efficacy for those species or strains [28]. The following probiotics were found in at least 2 trials to reduce: 1) NEC incidence: *Bifidobacterium lactis (B. lactis)* Bb-12 or B94; *Lactobacillus reuteri (L. reuteri)* ATCC 55730 or DSM 17938; LGG; the combination of *Bifidobacterium bifidum (B. bifidum)*, *B. infantis*, *Bifidobacterium longum (B. longum)* and *L. acidophilus;* the combination of *B. infantis* Bb-02, *B. lactis* Bb-12, and *Streptococcus thermophilus (S. thermophilus)* TH-4; the combination of *B.* 35624 and *LGG*; 2) LOS incidence: the combination of *B. bifidum*, *B. infantis*, *B. longum*, and *L. acidophilus* and the combination of *B. longum* R00175, *Lactobacillus helveticus (L.helveticus)* R0052, *L. rhamnosus* R0011, and *Saccharomyces boulardii (S. boulardii)* CNCM I-1079; 3) mortality: the combination of *B. bifidum* NCDO 1453 and *L. acidophilus* NCDO 1748. In 2019, a large metanalysis including 34 eligible studies and 9161 participants confirmed an advantage of probiotics in preventing NEC (3.54%) and gut-associated sepsis (15.59%), and in decreasing mortality (5.23%) in preterm infants [29]. Probiotic mixtures showed the highest advantage. The same conclusion on LOS is reported by a previous review [30]: pooled results from 37 RCTs and 9416 infants showed that probiotics significantly decreased the risk of LOS, with a number to treat of 44, in infants born < 37 WGA or < 2500 g. Beneficial effect in reducing LOS was reported by the analysis of studies including infants born < 32 WGA or < 1500 g. Subgroup analysis of extremely preterm infants (born < 28 WGA or < 1000 g) revealed no significant results. A recent single-centre retrospective observational study [31] compared 2 different populations: 469 versus 513 preterm infants born before or after the introduction of routine daily supplementation of *Lactobacillus* and *Bifidobacterium* for prophylaxis of NEC. NEC rate fell from 7.5 to 3.1% after the introduction of this policy (~ 55% RR reduction). The improvement affected all high-risk neonates irrespective of gestation age and feeding type. LOS rates also fell from 22.6 to 11.5%. Moreover, all-cause mortality rate decreased, although this was consistent with a trend over the study period. The positive effect of probiotics appeared greatest in the first 2 weeks after birth, suggesting that early postnatal colonization is crucial. The authors supported routine use of multi-species *Lactobacillus* and *Bifidobacterium* combination for preventing NEC. A further review of 30 articles, with a total number of 9522 preterm infants involved, of which 4812 receiving probiotics, showed that the supplementation significantly reduces the incidence of stage II-III NEC (RR = 0.55) [32]. Subgroup analysis indicated that mixed probiotics and *Lactobacillus* reduce the incidence of NEC (for mixed probiotics, RR = 0.39; for *Lactobacillus* RR = 0.53), while the individual use of *Bifidobacterium* or *Saccharomyces* did not have such effect. Similarly, probiotics’ supplementation significantly reduced the death rate (RR = 0.73), and subgroup analysis indicated that only mixed probiotics significantly reduce mortality (RR = 0.52), whereas *Lactobacillus*, *Bifidobacterium*, or *Saccharomyces* alone did not reach the same result [32]. More recently, after the last date of the search strategy of this review, ESPGHAN published an authoritative position paper on the use of probiotics in preterm infants [33]. The authors provide a conditional recommendation (with low certainty of evidence) to use either *LGG* or the combination of *B. infantis* Bb-02, *B. lactis* Bb-12, and *S. thermophilus* TH-4 in order to reduce NEC rates [33].

#### Safety

Albeit many trials do not report any adverse event, concerns regarding risk of probiotic supplementation have been raised [34, 35]. In particular, some recent cases of *Lactobacillus* or *Bifidobacterium* sepsis in infants receiving probiotics have been reported [36–46]. Most affected infants had severe diseases, as immunodeficiency [36] or short-gut syndrome [38]. Nevertheless, probiotic-associated sepsis, although rare and/or mild, should not be ignored. Probiotics are difficult to grow using standard culture media; bacteremia from probiotic strains may be under-recognized; centres routinely using probiotics must be confident that their laboratories can accurately identify these organisms and that empirical antibiotic therapy for LOS covers the probiotic strains locally in use [47]. Another safety concern is the probiotic shedding to someone who did not need supplementation. Probiotics could potentially improve neurodevelopmental outcome by reducing the incidence of NEC and LOS; alternatively, they could affect gut brain axis with unknown effects on neurological disorders in later life. Some safety studies concluded that oral probiotics do not affect neurodevelopment, growth and the risk of bronchopulmonary dysplasia [48–53], but further studies are needed.

#### Conclusions

Probiotic supplementation shows an overall advantage in preventing the incidence of NEC and gut-associated sepsis and decreasing mortality in preterm infants. Probiotics appear to be generally safe, but there are some reports about sepsis in preterm infants, potentially linked to supplementation. Also according to the last ESPGHAN position paper, the use of *LGG* or the combination of *B. infantis* Bb-02, *B. lactis* Bb-12, and *S. thermophilus* TH-4 showed some levels of evidence in reducing the incidence of NEC stage 2 and 3 and can therefore be recommended if all the safety conditions are met. Nevertheless, larger, well-designed trials are still needed to better understand the effect and safety of probiotics and to further solve the lack of clarity regarding the optimal dose, type of probiotic strain and timing of administration.

### Acute infectious Diarrhoea

Acute diarrhoea in childhood, usually defined by a decrease in the consistency of stools (loose or liquid) and/or an increase in the frequency of evacuations (> 3 episodes in 24 h), typically has an infectious aetiology and a relevant burden, either in high or low-income countries [54]. Historically, the treatment of AID has been the first field of application for probiotics [55, 56], and still today, represents the main indication for probiotic use in childhood. Due to the presence of compelling evidences showing an effect in reducing the duration of diarrhoea of about 24 h and the risk of severe AID, several guidelines recommend the use of probiotics worldwide, and a large number of children currently receives probiotics for the treatment of AID [57]. A recent study in about 4500 Japanese children reported 46.7% of probiotic prescription during an AID episode [58]. The ESPGHAN is currently updating the official recommendations for the use of selected probiotic strains in children with AID [54, 59]. In 2014, the EPGHAN graded as “moderate” the quality of evidences supporting the use of probiotics, meaning that further research was likely to produce more impact of evidence, according to the GRADE system. In the last years, new relevant evidences have become available.

#### Efficacy of selected probiotic strains

##### *Lactobacillus rhamnosus* GG

LGG is the best studied probiotic strain for the treatment of paediatric AID and received a strong recommendation by the ESPGHAN in 2014 [54]. However, in 2018, Schnadower and colleagues did not find efficacy in reduction of diarrhoea severity and duration in North-American children allocated to receive LGG in the largest randomized controlled trial published till now [60]. Those new and contradictory evidences rose a stimulating discussion among experts. The most recent metanalysis summarizing the data of 18 RCTs (4208 patients), including the latter North-American study, reported a reduction in duration of diarrhoea (mean difference (MD) -0.85 day, 95%CI − 1.15 to − 0.56), and hospitalization (MD − 1.22 day, 95%CI − 2.33 to − 0.10) [61], and confirmed a more evident efficacy in children living in European countries and/or receiving a daily LGG dose of ≥10^10^ CFU. However, notably, a further analysis including only five low-risk of bias RCTs found no effect of LGG on the duration of diarrhoea compared with controls (MD − 1.22 day 95%CI − 2.33 to − 0.10) [62].

Interestingly, comparing the effect size reported in metanalyses published in the last 10 years there is an apparent and progressive reduction of the efficacy of LGG on the duration of diarrhoea (the primary outcome of most studies) [61, 63, 64]. This may be seen as the effect of inclusion of new evidence, or (and) as the effect of variation in the enrolled population. It can be argued also that since LGG has a higher efficacy in rotavirus infection (and viral diarrhoea overall), the spreading of rotavirus immunization may potentially have an impact of the efficacy of LGG in immunized children and in those who benefit from the immunization’s “heard effect”. The effect of LGG has not been specifically studied in immunized children. Of note, the recent study by Schnadower and colleageues, although included a minority of children with rotaviral infection, reported a 67% rotavirus immunization coverage [60].

##### *Lactobacillus reuteri* DSM 17938

This strain received a weak recommendation from ESPGHAN in 2014 due to the small effect sizes of clinical evidence and methodological limitations of the included trials [54]. In the most recent metanalysis, compared with placebo or no treatment, *L. reuteri* reduced the duration of diarrhoea (MD − 0.87 days, 95%CI − 1.43 to − 0.31) and of hospitalization (MD − 0.54 days, 95%CI − 1.09 to 0.0) [65]. However, the paucity and inconsistency of data, and some methodological limitations should be taken into account before recommending a routine prescription of *L. reuteri* DSM 17938.

##### Saccharomyces boulardii

Multiple recent metanalyses consistently supported the use of the yeast *S. boulardii* in the treatment of children with AID, reporting a reduction in the duration of diarrhoea of about 1 day [66]. As a matter of fact, *S. boulardii* use is currently recommended at the same level of LGG in the management of acute gastroenteritis by the last ESPGHAN guidelines [53]. The more recent metanalysis published by Padayachee et al., and including only in-patients children affected by rotavirus infection, reported a significant reduction in the duration of diarrhoea (MD − 0.57 days, 95%CI − 0.83 to − 0.30) [67]. Also other recent studies, not included in the metanalysis, confirmed those trends [68] and, similarly, strength previous evidence on efficacy in the reduction of diarrhoea and of the length of hospital stay in children admitted for AID [69].

##### Bacillus clausii

Various probiotic formulations including different *Bacillus clausii* strains are currently available on the market. The strain O/C, SIN, N/R, and T is the most commonly studied and widely available in Italy. A recent meta-analysis by Ianiro et al. [70] reported a reduction in the duration of diarrhoea (MD − 9.12 h, 95%CI − 16.5 to − 1.75) and the duration of hospitalization (MD − 0.85 day, 95%CI − 1.56 to − 0.15). However, those pooled results should be looked with caution for several reasons that may affect their applicability in populations living in our setting: 1) the overall quality of evidence is very low, and none of the two “good quality” trials demonstrated any efficacy in terms of duration of diarrhoea, frequency of stools or duration of hospitalization, 2) most evidence have been published in non-indexed journals or as abstract 3) a significant publication bias (Egger’s test, *p* = 0.02) has been reported, 4) only one study has been conducted in a high-income European country, and did not demonstrate any efficacy in this population [71]. Further, more recent evidence reported safety and efficacy in children living in low-income countries [72].

#### Conclusions

Probiotics are largely used for the treatment of AID in childhood. Their use is based on studies of basic sciences that demonstrate a biological plausibility by one side, and evidence of clinical efficacy and safety by the other side. However, the evidence obtained in subjects with AID teaches us that the effect of probiotics depends on strain, dose, setting and patients’ conditions. According to the available evidence, only selected probiotic strains may be indicated, mainly *LGG*, *S. boulardii*, *L.reuteri*, while other combinations of Lactobacilli presented less convincing evidence (Table 1). The publication of two large randomized-controlled trials conducted in Northern America, one using LGG and the other using a combination of Lactobacilli (*Lactobacillus rhamnosus R0011 and L. helveticus R0052*) [60, 73], recently questioned the overall efficacy of probiotics in children with diarrhoea. Though a recent metanalysis including those results still found an efficacy of LGG on duration of AID [61], the impact of this evidence is not negligible and further research should address the role of LGG (as well as of other strains) in different setting and population (immunized or not for rotavirus).

**Table 1:** Summary of available evidence and clinical effects of selected probiotic strains commonly used for the treatment of acute infectious diarrhea

	***L. rhamnosus*** GG	***L. reuteri*** DSM 17938	***Saccharomyces boulardii***	***Bacillus clausii***
**Available Evidence**				
Highest level of evidence	Metanalysis	Metanalysis	Metanalysis	Metanalysis
Number of RCTs in children	16	4	22	7
**Investigational setting**				
Country Income ^a^	High Upper-middle Lower-middle	High Upper-middle	High Upper-middle Lower -middle	High Lower-middle
Inpatients/outpatients	Inpatients Outpatients	Inpatients Outpatients	Inpatients Outpatients	Inpatients Outpatients
Evidence in children living in Italy	Yes	Yes	Yes	Yes
**Outcomes**				
Duration of diarrhea	++	++	+++	+
Hospital admission	NA	–	NA	NA
Length of hospitalization	+++	+	+++	++
Healing within 48–72 h	+	NA	++	NA
Stool output	–	NA	NA	NA
Stool frequency	NA	–	+	–

^a^ level of income according to World Bank classification,

*NA* Not assessable,

(−) no effect, (+) minimal or borderline effect, (++) clinical effect, (+++) relevant and consistent clinical effect

### Allergic diseases

Paediatric allergic diseases such as atopic dermatitis (AD), food allergy (FA), allergic rhinitis and asthma represent major important public health issues in the western countries with growing prevalence in the last decades. Among different causative mechanisms the role of early gut microbiota development have been highlighted. Indeed, several data have shown the differences in allergic outcomes considering the effects of modifiable microbiota by external mechanisms, such as diet of the mother, drug assumption during pregnancy as well as infections and antibiotic use. All these factors associated with early factors such as the type of delivery, breastfeeding, the weaning and again the drug/antibiotic assumption during infancy have assumed growing evidences both for prevention and treatment of not-communicable and allergic diseases. Among different factors that can modulate the gut microbiota leading to preventive or therapeutic effects on paediatric allergic diseases, the probiotic effects are currently discussed with controversial results.

#### Efficacy of selected probiotic strains

##### *Lactobacillus rhamnosus* GG (LGG)

The preventive single administration of different strains, *LGG*, *B. lactis* Bb-12, *Lactobacillus paracasei* ST11 and *B. longum* BL999 was evaluated in 1 study [74]. Even though authors demonstrated that children at high risk receiving *LGG* perinatally tended to have decreased allergy prevalence (*p* < 0.047), the probiotic intervention gave no difference in growth or non-communicable disease prevalence [74]. Berni Canani and colleagues demonstrated that extensively hydrolysed casein formula containing the probiotic *LGG* not only can reduce the occurrence of other allergic manifestations in children with cow’s milk allergy (CMA) children but also hastens the development of oral tolerance [75]. Cabana et al., administering *LGG* supplementation for the first 6 months of life, investigated the cumulative incidence of eczema (primary end point) and asthma and rhinitis (secondary end points) in high-risk children. They were not able to demonstrate any preventive effect in the treated children [76]. Other authors investigated the effect of *LGG* plus vitamin D supplementation on the immunologic effectiveness of grass specific sublingual immunotherapy in children with allergy; they were able to show that probiotic supplementation gave better clinical and immunologic responses in children with allergic rhinitis who were treated with grass pollen immunotherapy [77].

#### Other single probiotic strains

In a two-centre randomized placebo-controlled trial of *Lactobacillus rhamnosus HN001* or *B. lactis HN019* taken daily from 35-week gestation to 6 months’ post-partum in mothers while breastfeeding and from birth to age 2 years in infants, authors showed that HN001 significantly protected against eczema development at 2, 4 and 6 years and atopic sensitization at 6 years [78]. There was no effect of HN019. At 11 years of age these data have been confirmed: HN001 was associated with a significant reduction in atopic sensitization, eczema. HN019 had no significant effect on these outcomes [78]. In another study *Lactobacillus plantarum* IS-10506 supplementation given twice daily for 14 weeks in 22 children with mild to moderate AD, reduced SCORAD and levels of inflammatory markers [79]. *L. reuteri* DSM 17938 plus vitamin D3 in 32 children with mild persistent asthma was demonstrated to be effective in reducing markers of bronchial inflammation [80].

#### Mixture of strains

In a RCT a mixture of strains, (*B. lactis CECT 8145*, *B. longum CECT 7347*, and *Lactobacillus casei CECT 9104*) were able to produce a mean reduction in the SCORAD index in the probiotic group with a significant reduction in the use of topical steroids to treat flares [81]. Pregnant women (*n* = 1223) carrying a child at a high risk of allergy were randomized to receive a mixture of probiotics (*LGG* and *LC705*, *B. breve Bb99* and *Propionibacterium freudenreichii*) or placebo in a double-blind manner from 36 weeks of gestation until birth. Their infants received the same product for the first 6 months. In the whole cohort, there were no statistically significant differences in the diagnosis of allergic diseases. In a post-hoc analysis made in Caesarean-delivered subgroup, allergy was reported in 41.5% of the probiotic group and 67.9% of the placebo group [82]. Bifidobacterium mixture (*B. longum BB536*, *B. infantis M-63*, *B. breve M-16 V*) treatment for 4 weeks in children with seasonal allergic rhinitis determined significant improvement of symptoms (*p* < 0.005), and quality of life (*p* < 0.001). Placebo group had worsening of symptoms [83]. In a double-blinded trial from 36-week gestation until 3 months postpartum 415 pregnant women were randomized to receive a probiotic blend containing *LGG*, *L. acidophilus La-5* and *Bifidobacterium animalis subsp. lactis Bb-12* or placebo. At 6 years, while there was a trend towards a lower cumulative incidence of AD in the probiotic group, prevalence of asthma and atopic sensitization were not significantly affected by the probiotic regime [84]. *Lactobacillus paracasei* and *Lactobacillus fermentum* alone or in a mixture were evaluated for the effects in 220 children affected by AD. Children who received for 3 months single probiotic strains or mixture showed lower SCORAD scores than placebo; this difference persisted even at 4 months after discontinuing the supplementation [85]. *Lactobacillus rhamnosus* in children aged 4–48 months with AD for 8 weeks reduced the clinical scores. However, no significant difference between groups was noted in terms of amount of topical corticosteroids used or overall symptom-free time [86].

#### Metanalyses and reviews

A metanalysis performed by the World Allergy Organization demonstrated that probiotics used by pregnant women or breastfeeding mothers and/or given to infants can reduce the risk of eczema in infants [87]; however, the certainty of the evidence was low. No effect was observed for the prevention of other allergic conditions [87]. Huang, evaluating SCORAD values, demonstrated more favourable results using probiotics over controls. However, the results were so controversial that data from Europe revealed no effect of probiotics on SCORAD, whereas significantly lower SCORAD values were reported in Asia. No effect was observed with *LGG* and *Lactobacillus plantarum*, whereas some effects were observed with *Lactobacillus fermentum* and *Lactobacillus salivarius* and a mixture of different strains [88]. Similar controversial effects were obtained by a review investigating the microbial modulations of the intestinal microbiome with pre- and/or probiotics used in AD management [88]. Clinical studies showed that some dietary interventions with pre- and/or probiotics were beneficial, but the great heterogeneity between studies was high, making it clear that focused RCTs are needed to understand the potential role and underlying mechanism of dietary interventions in children with AD [89]. Zhang et al. evaluated seventeen trials involving 2947 infants indicating that probiotics administered pre-natally and post-natally could reduce the risk of atopy and the risk for food hypersensitivity. When probiotics were administered either only pre-natally or only post-natally, no effect on atopic diseases was observed [90]. Finally, Zuccotti et al. in 2015 evaluated in a meta-analysis the effects of probiotics in the prevention of atopic diseases in infants [91]. Infants treated with probiotics had a significantly lower RR for eczema, especially those supplemented with a mixture of probiotics. No significant difference in terms of prevention of asthma was observed in the considered trials [91].

#### Conclusions

The effects of probiotic administration for prevention/treatment of allergic diseases are still so controversial that no firm recommendation can be made at this stage. Differences in strain specificity, timing of administration, and length of the therapy are all contributing to diversify the metanalysis conclusions. Therefore, further strain specific studies are necessary in order to clarify the role of probiotics in modulating the allergic manifestations.

### Functional gastrointestinal disorders

Functional Gastrointestinal Disorders (FGIDs) include a wide range of GI disorders that cannot be explained by structural or biochemical abnormalities. Diagnosis is based on the Rome IV criteria [92, 93]. Clinical expression varies according on the age of appearance, though different sets of criteria are addressed to neonates/toddlers and children/adolescents. FGIDs occurring during the first years of life include: Infant regurgitation, Infant rumination syndrome, Cyclic vomiting syndrome, Infant colic, Functional diarrhoea, Infant dyschezia, Functional constipation. Among children and adolescents FGIDs are grouped into three main classes: functional nausea and vomiting disorders, functional abdominal pain disorders and functional defecation disorders [92, 93]. The reported prevalence ranges from 27 to 38% in neonates and toddlers [94], 9.9 to 29% in older children [95]. Whereas alterations in gut microbiome may have a causative role in the pathogenesis of the FGIDs, probiotics’ supplementation has been proposed as a possible option in various FGDIs management.

#### Infant colic

Infant colic is described as recurrent and prolonged periods of crying, fussing, or irritability without obvious cause in infants younger than 5 months of age [92]. It is reported to be one of the most prevalent FGIDs in the first year of life, affecting 20% of infants less than 3 months of age, especially between 2 weeks to 4 months of age. The aetiology is related to different factors including feeding difficulties, dysmotility, hormone alterations and behavioural factors [92]. Management is traditionally based on changes in feeding, soothing techniques and parental support [92]. Since 2007, several RCTs suggested probiotics, particularly *L. reuteri*, as a potential intervention (Table 2). Supplementation with this strain seems to be more effective in breastfed infant. A recently published IPMDA (individual participant data metanalysis) [99] on *L. reuteri DSM17398* effectiveness demonstrated that supplementation with this strain lead to reduction in cry/fuss time. The pooled analysis of four RCTs [96–98, 100] published between 2007 and 2015, showed that infants receiving *L. reuteri* had a significant reduction of the crying and/ or fussing duration, with a number needed to treat of 2.6 (95% CI, 2.0 to 3.6) in breastfed infants. All included studies were double-blind randomized placebo-controlled trials of high quality, the intervention groups received the same probiotic (*L. reuteri* DSM17398) manufactured by the same company, in the same dose, (0.2 × 10^8^ CFU per drop, 5 drops orally per day), with the control groups all receiving the same placebo (maltodextrin in oil suspension). Notably diagnoses were based on modified Wessel’s criteria (crying or fussy/gassy episodes ≥3 h/day for ≥3 days/7 days) and success treatment on parents’ reports. Since the majority of infants included in the studies were breastfed, this result cannot be extended to all infants. As matter of fact the only study [98] that evaluated formula-fed infants found no significant difference between treated and control group. *L. reuteri* appears to be a potential treatment option for breastfed infants with colic.

**Table 2:** Summary of the studies reporting the effects of *Lactobacillus reuteri* for the treatment of Infant Colic

Author and year	Intervention	Control	Treatment duration	Follow up	Type of feeding
**Savino F et al. 2010** [96]	*Lactobacillus reuteri* DSM 17938 10^8^ CFU (5 drops)	Placebo (identical formulation but without live bacteria)	21 days	No follow up	Exclusive breast-feeding
**Szajewska H et al. 2013** [64]	*Lactobacillus reuteri* DSM 17938 10^8^ CFU (5 drops)	Placebo (identical formulation but without live bacteria)	21 days	8 days	Exclusive or predominat (> 50%) breast-feeding
**Chau K et al. 2015** [97]	*Lactobacillus reuteri* DSM 17938 10^8^ CFU (5 drops)	Placebo (same excipient ingredients but without live bacteria)	21 days	No follow up	Exclusive breast-feeding
**Sung V et al. 2014** [98]	*Lactobacillus reuteri DSM 17938* 10^8^ CFU *(5 drops)*	Placebo (maltodextrin in the same oil suspension)	1 month	5 months	Formula feeding, breast feeding

#### Irritable bowel syndrome

Irritable bowel syndrome (IBS) is characterized by recurrent abdominal pain and changes in stool form or frequency without any organic cause [92, 93]. According to the predominant bowel type, 3 subtypes are described: diarrhoea, constipation or mixed (IBS-M). It is the commonest FGID in children, with prevalence ranging from 1.2 to 2.9% [93]. Based on Rome IV criteria abdominal pain associated with defecation or a change in frequency or form of stool must be present 4 days per months for at least 2 months [93]. IBS is considered a brain-gut axis disorder involving visceral hypersensitivity, mucosal pro-inflammatory cytokines, altered mucosal and immune function and disturbance in gut microbiota [101]. Gut microbiota alterations are thought to be relevant in IBS pathogenesis [102]. As a matter of fact, studies on rodents have shown that the administration of probiotics leads to a reduction in visceral pain sensitization [103, 104]. In addition to these preliminary data, small intestinal bacterial overgrowth and symptoms developing after gastrointestinal infection share some features with IBS clinical presentation suggesting a possible causative role of gut microbiome [105]. In a Cochrane metanalysis on dietary intervention for recurrent abdominal pain (RAP), the subgroup analysis on children with IBS (Rome III criteria), including 4 RCTs and 344 children, showed that OR for pain improvement at 0 to 3 months’ post-intervention was 3.01 (95% CI 1.77 to 5.13, *P* < 0.001, I^2^ = 21%; P for heterogeneity = 0.29), with a number needed to treat (NTT) of four [106]. Using GRADE the quality of the evidence was considered moderate. Two studies from Bauserman M et al. [107] and Francavilla et al. [108] evaluated the efficacy of the mono-strain probiotic, *LGG*, even if in different concentration (Bauserman M et al. 2005 10^10^ bacteria, Francavilla R et al. 3X 10^9^ colony-forming units). Bauserman M and colleagues did not find any effect in terms of pain reduction and number of responders [107]. Guandalini et al. [109] tested *VSL#3*, a combination of seven probiotic bacteria, while Giannetti et al. used a mixture of 3 *Bifidobacteria* (namely, 3 billions *B. longum* BB536, 1 billion of *B. infantis* M-63, and 1 billion of *B. breve* M-16 V [110]. A recently published systematic review [111] confirmed an overall beneficial effect of probiotic in children suffering from IBS. Particularly, Gawronska et al. 2007 found that 4 weeks after enrolment 33% of patients (6/18) in the study group, using *LGG* (3 X 10^9^ CFU) referred no pain, with a NTT of four [112]. Given the small sample size this result should be evaluated with caution. Finally, Kianifar H et al. demonstrated a statistically significant difference in pain severity in the probiotic group, with *LGG* dosage of 1 × 10^10^ CFU/ml, measured as reduction in the pain severity scale (Likert scale) [113] (Table 3).

**Table 3:** Summary of the studies reporting the effects of different probiotics strains for the treatment of Irritable Bowel Syndrome and Functional Abdominal Pain-Not Otherwise

Author and year	Intervention	Control	Treatment duration	Follow up
**Bauserman M et al. 2005** [107]	*Lactobacillus GG* 10^10^ bacteria twice per day	Placebo (inulin)	6 weeks	No follow up after treatment period
**Francavilla R et al. 2010** [108]	*Lactobacillus GG* 3X10^9^ CFU twice per day	Placebo	8 weeks	8 weeks
**Guandalini S et al. 2010** [109]	VSL#3 1 sachet once per day 4-11 years Twice per day 12–18 years	Placebo	6 weeks	6 weeks ^a^
**Giannetti e et al. 2017** [110]	*Bifidobaacterium longum* BB536 3 billions, *Bifidobacterium infantis* M-63 1 billion, *Bifidobacterium breve* M-16 V 1 billion	Placebo	6 weeks	6 weeks ^a^
**Gawrońska A et al. 2007** [112]	*Lactobacillus GG* 3X10^9^ twice per day	Placebo	4 weeks	No follow up after treatment period
**Kianifar H et al** **2015** [113]	*Lactobacillus GG* 1X10^10^ CFU/ml twice per day	Placebo	4 weeks	No follow up after treatment period

^a^ At the completion of the 6 weeks, no preparation was administered for 2 weeks. Then each patient was switched to the other group and followed for 6 weeks

#### Functional abdominal pain

Functional Abdominal Pain-Not Otherwise (FAP-NOS), according to Rome IV criteria, is characterized by episodic or continuous abdominal pain that does not occur solely during physiologic events occurring 4 times/month for at least 2 months before diagnosis [93]. Few papers on the use probiotics for this subtype of FADP have been published. Jadrešin O et al. [114] demonstrated that administration of *L. reuteri* DSM 17938 is associated with a reduction of the intensity and frequency of pain in population study including patients with FAP and IBS. Maragkoudaki M et al. [115] found that in a paediatric cohort *L. reuteri* DSM 17938, at a dose of 2 X 10^8^ CFU was not different from placebo in reducing the frequency and intensity of the abdominal pain episodes. By contrast in the intervention arm a reduction in pain relieving drugs was registered, even if not statistically significant. Weizman Z et al. [116] administration of *L. reuteri* DSM 17938, at a dose of 1X 10^8^ CFU reduced frequency and intensity of abdominal pain in children with FAP at 4 weeks following supplementation, and during a 4-week follow up period when no probiotics was used. Romano C et al. [117] showed that oral supplementation with *L. reuteri* DSM 17938, 2 × 10^8^ CFU in children with FAP led to a significant reduction in the reported intensity but not the frequency of abdominal pain, both during and after cessation of administration of the probiotic. In all of these studies FAP diagnosis was made according Rome III criteria, Symptoms were evaluated using Wong-Baker FACES Pain Rating Scale (Table 3).

#### Conclusions

Despite probiotics appears to be a promising treatment for the treatment of FGDIs subtypes, the overall quality and quantity of evidences are relatively weak and therefore more studies with robust design are needed to evaluate efficacy of either mono- or multistrain supplementation, and the most appropriate dose.

## Conclusive remarks

Several limitations, including the use of different strains, various concentrations of the same strain, heterogeneity in study designs and small sample size, strongly limit the evidences of efficacy of probiotics in paediatric diseases. Despite these limitations, the use of *LGG* or the combination of *B. infantis* Bb-02, *B. lactis* Bb-12, and *S. thermophilus* TH-4 can be recommended to reduce the incidence of NEC stage 2 and 3. Nevertheless, further well-designed studies are needed to better identify the strains to be used and even clarify the safety issues. In the setting of acute infectious diarrhoea, regardless the last published papers, the use of *LGG and S. Boulardii* for the reduction of the duration is still supported by convincing evidences. Differently, no conclusions can be made in allergic diseases, due to the conflicting data and the different strains used. A higher level of standardization in terms of doses, strains and outcomes is warranted in order to better answer the question whether probiotics may help the process of immune tolerance induction. Finally, the use of probiotics for the treatment of FGIDs, although supported by a strong rationale, did not lead to the expected results. Except for the administration of *L. reuteri* in breastfed children with infantile colic, up to date the administration of probiotics cannot be recommended for the care of FGIDs. In particular, despite few trials with different designs and outcomes, probiotics failed to show efficacy in the treatment of pain related disorders such as IBS or FAP-NOS.

## Data Availability

Not applicable.

## Abbreviations

AD: Atopic dermatitis
AID: Acute infectious diarrhoea
*B. bifidum*: *Bifidobacterium bifidum*
*B.infantis*: *Bifidobacterium infantis*
*B.breve*: *Bifidobacterium breve*
*B.longum*: *Bifidobacterium longum*
CFU: Colony-forming units
CMA: Cow’s milk allergy
EFSA: European food safety authority
ELBW: Extremely low birth weight
ESPGHAN: European society of pediatric gastroenterology, hepatology and nutrition
FAP-NOS: Functional abdominal pain-not otherwise
FGIDs: Functional gastrointestinal disorders
FA: Food allergy
IPMDA: Individual participant data metanalysis
IBS: Irritable bowel syndrome
ISAPP: International scientific association for probiotics and prebiotics
HM: Human milk
IUGR: Intrauterine growth restriction
*L. acidophilus*: *Lactobacillus acidophilus*
*L. bifidum*: *Lactobacillus bifidum*
*L.helveticus*: *Lactobacillus helveticus*
*L. reuteri*: *Lactobacillus reuteri*
*LGG*: *Lactobacillus rhamnosus GG*
LOS: Late onset sepsis
NMA: Network Metanalysis
NEC: Necrotizing enterocolitis
NICU: Neonatal intensive care unit
RAP: Recurrent abdominal pain
RCTs: Randomized controlled trials
*S. boulardii*: *Saccharomyces boulardii*
*S. thermophilus*: *Streptococcus thermophilus*
VLBW: Very low birth weight
WGA: Weeks of gestational age

## References

[1] Hill C, Guarner F, Reid G, Gibson GR (2014). Expert consensus document. The international scientific Association for Probiotics and Prebiotics consensus statement on the scope and appropriate use of the term probiotic. Nat Rev Gastroenterol Hepatol.

[2] Food and Agricultural Organization of the United Nations and World Health Organization. Health and nutritional properties of probiotics in food including powder milk with live lactic acid bacteria: World Health Organization; 2001. http://www.who.int/foodsafety/publications/fs_management/en/probiotics.pdf.

[3] Bertazzoni E, Donelli G, Midtvedt T, Nicoli J, Sanz Y (2013). Probiotics and clinical effects: is the number what counts?. J Chemother.

[4] Health Canada. Accepted claims about nature of probiotic microorganisms in food. Health Canada. http://www.inspection.gc.ca/food/%20labelling/food-labelling-for-industry/health-claims/eng/1392834838383/%201392834887794?chap=9.

[5] Federal Regulation of Probiotics: An Analysis of the. Existing Regulatory Framework and Recommendations for. Alternative Frameworks. NIH Grant Number:5R01HG00517102;2016.http://www.law.umaryland.edu/ProbioticsWhitePaper.

[6] Health Food Regulatory System in Japan. Available at: https://food.chemlinked.com/chempedia/health-food-regulatory-system-japan.

[7] Food Products Directive and Regulation. Regulation 178/2002/EC; directive 2000/13/EU.

[8] FAQ on Nutrition and Health Claims, EFSA, http://www.efsa.europa. eu/en/fasq/faqnutrition.htm.

[9] Ministero della Salute, Commissione unica per la nutrizione e la dietetica. Guidelines on probiotics and prebiotics. *Ministero della Salute* [online], http://www.salute.gov.it/imgs/C_17_pubblicazioni_1016_allegato.pdf (2013).

[10] Kolaček S, Hojsak I, Berni Canani R (2017). ESPGHAN working Group for Probiotics and Prebiotics. Commercial probiotic products: a call for improved quality control. A position paper by the ESPGHAN working Group for Probiotics and Prebiotics. J Pediatr Gastroenterol Nutr.

[11] US Food and Drug Administration. Current good manufacturing practice in manufacturing, Packaging, Labelling, or Holding Operation for Dietary Supplements, 2007.

[12] McFarland LV (2015). From yaks to yogurt: the history, development, and current use of probiotics. Clin Infect Dis.

[13] Hojsak I, Fabiano V, Pop TL (2018). Guidance on the use of probiotics in clinical practice in children with selected clinical conditions and in specific vulnerable groups. Acta Paediatr.

[14] Szajewska H (2016). What are the indications for using probiotics in children?. Arch Dis Child.

[15] Zheng J, Wittouck S, Salvetti E, et al. A taxonomic note on the genus *Lactobacillus*: Description of 23 novel genera, emended description of the genus *Lactobacillus* Beijerinck 1901, and union of *Lactobacillaceae* and *Leuconostocaceae*. Int J Syst Evol Microbiol. 2020. 10.1099/ijsem.0.004107 published online ahead of print, 2020 Apr 15. 10.1099/ijsem.0.004107 32293557

[16] Neu J, Walker WA (2011). Necrotizing enterocolitis. N Engl J Med.

[17] Berrington JE, Hearn RI, Bythell M, Wright C, Embleton ND (2012). Deaths in preterm infants: changing pathology over 2 decades. J Pediatr.

[18] Berrington JE, Stewart CJ, Cummings SP, Embleton ND (2014). The neonatal bowel microbiome in health and infection. Curr Opin Infect Dis.

[19] Cassir N, Benamar S, Khalil JB (2015). Clostridium butyricum strains and dysbiosis linked to necrotizing enterocolitis in preterm neonates. Clin Infect Dis.

[20] Morrow AL, Lagomarcino AJ, Schibler KR (2013). Early microbial and metabolomics signatures predict later onset of necrotizing enterocolitis in preterm infants. Microbiome.

[21] AlFaleh K, Anabrees J. Probiotics for prevention of necrotizing enterocolitis in preterm infants. Cochrane Database Syst Rev. 2014;4. 10.1002/14651858.CD005496.pub4 24723255

[22] Adams M, Bassler D, Darlow BA, et al. International Network for Evaluating Outcomes (iNeo) of Neonates. Preventive strategies and factors associated with surgically treated necrotising enterocolitis in extremely preterm infants: an international unit survey linked with retrospective cohort data analysis. BMJ Open. 2019;9:e031086. 10.1136/bmjopen-2019-031086 PMC6797308 31615799

[23] Aceti A, Gori D, Barone G (2015). Italian Society of Neonatology Probiotics for prevention of necrotizing enterocolitis in preterm infants: systematic review and meta-analysis. Ital J Pediatr.

[24] Costeloe K, Hardy P, Juszczak E (2016). Bifidobacterium breve BBG-001 in very preterm infants: a randomised controlled phase 3 trial. Lancet.

[25] Uberos J, Aguilera-Rodríguez E, Jerez-Calero A, Molina-Oya M, Molina-Carballo A, Narbona-López E (2017). Probiotics to prevent necrotising enterocolitis and nosocomial infection in very low birth weight preterm infants. Br J Nutr.

[26] Aceti A, Maggio L, Beghetti I (2017). On behalf of the Italian Society of Neonatology. Probiotics prevent late-onset sepsis in human milk-fed, very low birth weight preterm infants: systematic review and meta-analysis. Nutrients.

[27] Dermyshi E, Wang Y, Yan C, Hong W, Qiu G, Gong X, Zhang T (2017). The "Golden age" of probiotics: a systematic review and meta-analysis of randomized and observational studies in preterm infants. Neonatology.

[28] van den Akker CHP, van Goudoever JB (2018). ESPGHAN working Group for Probiotics, Prebiotics & Committee on nutrition. Probiotics for preterm infants: a strain-specific systematic review and network meta-analysis. J Pediatr Gastroenterol Nutr.

[29] Bi LW, Yan BL, Yang QY, Li MM, Cui HL (2019). Probiotic strategies to prevent necrotizing enterocolitis in preterm infants: a meta-analysis. Pediatr Surg Int.

[30] Rao SC, Athalye-Jape GK, Deshpande GC, Simmer KN, Patole SK. Probiotic supplementation and late-onset Sepsis in preterm infants: a meta-analysis. Pediatrics. 2016;137. 10.1542/peds.2015-3684 26908700

[31] Robertson C, Savva GM, Clapuci R, et al. Incidence of necrotising enterocolitis before and after introducing routine prophylactic Lactobacillus and Bifidobacterium probiotics. Arch Dis Child Fetal Neonatal Ed. 2020;105:380-6. 10.1136/archdischild-2019-317346 PMC7363787 31666311

[32] Jiang T, Zhang H, Xu X, Li H, Yang J (2019). Mixed probiotics decrease the incidence of stage II-III necrotizing enterocolitis and death: a systematic review and meta-analysis. Microb Pathog.

[33] van den Akker CHP, van Goudoever JB, Shamir R (2020). Probiotics and preterm infants: a position paper by the European Society for Paediatric Gastroenterology Hepatology and Nutrition Committee on nutrition and the European Society for Paediatric Gastroenterology Hepatology and Nutrition Working Group for probiotics and prebiotics. J Pediatr Gastroenterol Nutr.

[34] Jacobs SE, Tobin JM, Opie GF (2013). Probiotic effects on late-onset sepsis in very preterm infants: a randomized controlled trial. Pediatrics.

[35] Trivić I, Mlakar AS, Hojsak I (2019). The role of probiotics in the prevention of necrotizing Enterocolitis. Curr Pediatr Rev.

[36] Land MH, Rouster-Stevens K, Woods CR, Cannon ML, Cnota J, Shetty AK (2005). Lactobacillus sepsis associated with probiotic therapy. Pediatrics.

[37] Hammerman C, Bin-Nun A, Kaplan M (2006). Safety of probiotics: comparison of two popular strains. BMJ.

[38] Kunz AN, Noel JM, Fairchok MP (2004). Two cases of Lactobacillus bacteremia during probiotic treatment of short gut syndrome. J Pediatr Gastroenterol Nutr.

[39] Ohishi A, Takahashi S, Ito Y, Ohishi Y (2010). Bifidobacterium septicemia associated with postoperative probiotic therapy in a neonate with omphalocele. J Pediatr.

[40] Zbinden A, Zbinden R, Berger C, Arlettaz R (2015). Case series of Bifidobacterium longum bacteremia in three preterm infants on probiotic therapy. Neonatology.

[41] Bertelli C, Pillonel T, Torregrossa A (2015). Bifidobacterium longum bacteremia in preterm infants receiving probiotics. Clin Infect Dis.

[42] Jenke A, Ruf EM, Hoppe T, Heldmann M, Wirth S (2012). Bifidobacterium septicaemia in an extremely low-birth weight infant under probiotic therapy. Arch Dis Child Fetal Neonatal Ed.

[43] Brecht M, Garg A, Longstaff K, Cooper C, Andersen C (2016). Lactobacillus Sepsis following a laparotomy in a preterm infant: a note of caution. Neonatology.

[44] Esaiassen E, Cavanagh P, Hjerde E, Simonsen GS, Støen R, Klingenberg C (2016). Bifidobacterium longum subspecies infantis bacteremia in 3 extremely preterm infants receiving probiotics. Emerg Infect Dis.

[45] Molinaro M, Aiazzi M, La Torre A, Cini E, Banfi R (2016). Lactobacillus Rhamnosus sepsis in a preterm infant associated with probiotic integrator use: a case report. Recenti Prog Med.

[46] Celis Castañeda LA, Morales Camacho WJ, Durán Ochoa NM (2019). Sepsis due to Lactobacillus reuteri in an extreme preterm newborn: case report. Arch Argent Pediatr.

[47] Fleming PF, Berrington JE, Jacobs SE (2019). Addressing safety concerns of probiotic use in preterm babies. Early Hum Dev.

[48] Sari FN, Eras Z, Dizdar EA (2012). Do oral probiotics affect growth and neurodevelopmental outcomes in very low-birth-weight preterm infants?. Am J Perinatol.

[49] Jacobs SE, Hickey L, Donath S (2017). ProPremsStudy Groups. Probiotics, prematurity and neurodevelopment: follow-up of a randomised trial. BMJ Paediatr Open.

[50] Chou IC, Kuo HT, Chang JS (2010). Lack of effects of oral probiotics on growth and neurodevelopmental outcomes in preterm very low birth weight infants. J Pediatr.

[51] Romeo MG, Romeo DM, Trovato L (2011). Role of probiotics in the prevention of the enteric colonization by Candida in preterm newborns: incidence of late-onset sepsis and neurological outcome. J Perinatol.

[52] Hays S, Jacquot A, Gauthier H (2016). Probiotics and growth in preterm infants: a randomized controlled trial, PREMAPRO study. Clin Nutr.

[53] Villamor-Martínez E, Pierro M, Cavallaro G, Mosca F, Kramer B, Villamor E. Probiotic supplementation in preterm infants does not affect the risk of bronchopulmonary dysplasia: a meta-analysis of randomized controlled trials. Nutrients. 2017; 9. pii: E1197. 10.3390/nu9111197 PMC5707669 29088103

[54] Guarino A, Ashkenazi S, Gendrel D (2014). European Society for Pediatric Gastroenterology, Hepatology, and nutrition/European Society for Pediatric Infectious Diseases evidence-based guidelines for the management of acute gastroenteritis in children in Europe: update 2014. J Pediatr Gastroenterol Nutr.

[55] Guarino A, Canani RB, Spagnuolo MI (1997). Oral bacterial therapy reduces the duration of symptoms and of viral excretion in children with mild diarrhea. J Pediatr Gastroenterol Nutr.

[56] Guandalini S, Pensabene L, Zikri MA (2000). Lactobacillus GG administered in oral rehydration solution to children with acute diarrhea: a multicenter European trial. J Pediatr Gastroenterol Nutr.

[57] Lo Vecchio A, Dias JA, Berkley JA (2016). Comparison of recommendations in clinical practice guidelines for acute gastroenteritis in children. J Pediatr Gastroenterol Nutr.

[58] Okubo Y, Miyairi I, Michihata N (2019). Recent prescription patterns for children with acute infectious diarrhea. J Ped Gastroenterol Nutr.

[59] Szajewska H, Guarino A, Hojsak I (2014). European Society for Pediatric Gastroenterology, Hepatology, and nutrition. Use of probiotics for management of acute gastroenteritis: a position paper by the ESPGHAN working group for probiotics and prebiotics. J Pediatr Gastroenterol Nutr.

[60] Schnadower D, Tarr PH, Casper C, Gorelick MH (2018). Lactobacillus rhamnosus GG versus placebo for acute gastroenteritis in children. N Engl J Med.

[61] Szajewska H, Kołodziej M, Gieruszczak-Białek D, Skórka A, Ruszczyński M, Shamir R (2019). Systematic review with meta-analysis: Lactobacillus Rhamnosus GG for treating acute gastroenteritis in children - a 2019 update. Aliment Pharmacol Ther.

[62] Schnadower D, Tarr PI, Freedman SB. Letter: Lactobacillus rhamnosus GG offers no benefit over placebo in children with acute gastroenteritis. Aliment Pharmacol Ther. 2019;50:620–2. 10.1111/apt.15418 31414542

[63] Allen SJ, Martinez EG, Gregorio GV, Dans LF (2010). Probiotics for treating acute infectious diarrhoea. Cochrane Database Syst Rev.

[64] Szajewska H, Skorka A, Ruszczynski M (2013). Meta-analysis: Lactobacillus GG for treating acute gastroenteritis in children--updated analysis of randomised controlled trials. Aliment Pharmacol Ther.

[65] Patro-Gołąb B, Szajewska H. Systematic review with meta-analysis: Lactobacillus reuteri DSM 17938 for treating acute gastroenteritis in children. An Update. Nutrients. 2019;11:2762. 10.3390/nu11112762 PMC6893691 31739457

[66] Feizizadeh S, Salehi-Abargouei A, Akbari V (2014). Efficacy and safety of Saccharomyces boulardii for acute diarrhea. Pediatrics.

[67] Padayachee M, Visser J, Viljoen E (2019). Efficacy and safety of Saccharomyces boulardii in the treatment of acute gastroenteritis in the paediatric population: a systematic review. S Afr J Clin Nutr.

[68] Das S, Gupta PK, Das RR (2016). Efficacy and safety of Saccharomyces boulardii in acute rotavirus diarrhea: double blind randomized controlled trial from a developing country. J Trop Pediatr.

[69] Dinleyici EC, Eren M, Ozen M (2012). Effectiveness and safety of Saccharomyces boulardii for acute infectious diarrhea. Expert Opin Biol Ther.

[70] Ianiro G, Rizzatti G, Plomer M, et al. *Bacillus clausii* for the treatment of acute diarrhea in children: a systematic review and meta-analysis of randomized controlled trials. Nutrients. 2018; 10. pii: E1074. 10.3390/nu10081074 PMC6116021 30103531

[71] Canani RB, Cirillo P, Terrin G (2007). Probiotics for treatment of acute diarrhoea in children: randomised clinical trial of five different preparations. BMJ.

[72] de Castro JA, Villa-Real Guno MJ, Perez MO. Bacillus clausii as adjunctive treatment for acute community-acquired diarrhea among filipino children: a large-scale, multicenter, open-label study (CODDLE). Trop Dis Travel Med Vaccines. 2019;5:14. 10.1186/s40794-019-0089-5 PMC6651909 31367461

[73] Freedman SB. Williamson-Urquhart S, Farion KJ, et al. PERC PROGUT trial group. Multicenter trial of a combination probiotic for children with gastroenteritis. N Engl J Med. 2018;379:2015–26. 10.1056/NEJMoa1802597 30462939

[74] Lundelin K, Poussa T, Salminen S, Isolauri E (2017). Long-term safety and efficacy of perinatal probiotic intervention: evidence from a follow-up study of four randomized, double-blind, placebo-controlled trials. Pediatr Allergy Immunol.

[75] Berni Canani R, Di Costanzo M, Bedogni G (2017). Extensively hydrolyzed casein formula containing *Lactobacillus rhamnosus* GG reduces the occurrence of other allergic manifestations in children with cow's milk allergy: 3-year randomized controlled trial. J Allergy Clin Immunol.

[76] Cabana MD, McKean M, Caughey AB, et al. Early probiotic supplementation for eczema and asthma prevention: a randomized controlled trial. Pediatrics. 2017; 140. pii: e20163000. 10.1542/peds.2016-3000 PMC5574725 28784701

[77] Jerzynska J, Stelmach W, Balcerak J (2016). Effect of Lactobacillus rhamnosus GG and vitamin D supplementation on the immunologic effectiveness of grass-specific sublingual immunotherapy in children with allergy. Allergy Asthma Proc.

[78] Wickens K, Barthow C, Mitchell EA (2018). Effects of Lactobacillus rhamnosus HN001 in early life on the cumulative prevalence of allergic disease to 11 years. Pediatr Allergy Immunol.

[79] Prakoeswa CRS, Herwanto N, Prameswari R (2017). Lactobacillus plantarum IS-10506 supplementation reduced SCORAD in children with atopic dermatitis. Benef Microbes.

[80] Miraglia Del Giudice M, Maiello N (2016). Lactobacillus reuteri DSM 17938 plus vitamin D(3) as ancillary treatment in allergic children with asthma. Ann Allergy Asthma Immunol.

[81] Navarro-López V, Ramírez-Boscá A, Ramón-Vidal D (2018). Effect of Oral Administration of a Mixture of probiotic strains on SCORAD index and use of topical steroids in young patients with moderate atopic dermatitis: a randomized clinical trial. JAMA Dermatol.

[82] Kallio S, Kukkonen AK, Savilahti E, Kuitunen M (2019). Perinatal probiotic intervention prevented allergic disease in a caesarean-delivered subgroup at 13-year follow-up. Clin Exp Allergy.

[83] Miraglia Del Giudice M, Indolfi C (2017). Bifidobacterium mixture (B longum BB536, B infantis M-63, B breve M-16V) treatment in children with seasonal allergic rhinitis and intermittent asthma. Ital J Pediatr.

[84] Simpson MR, Dotterud CK, Storrø O, Johnsen R, Øien T (2015). Perinatal probiotic supplementation in the prevention of allergy related disease: 6 year follow up of a randomised controlled trial. BMC Dermatol.

[85] Wang IJ, Wang JY (2015). Children with atopic dermatitis show clinical improvement after Lactobacillus exposure. Clin Exp Allergy.

[86] Wu YJ, Wu WF, Hung CW (2017). Evaluation of efficacy and safety of Lactobacillus rhamnosus in children aged 4-48 months with atopic dermatitis: an 8-week, double-blind, randomized, placebo-controlled study. J Microbiol Immunol Infect.

[87] Cuello-Garcia CA, Brożek JL, Fiocchi A (2015). Probiotics for the prevention of allergy: a systematic review and meta-analysis of randomized controlled trials. J Allergy Clin Immunol.

[88] Huang R, Ning H, Shen M, Li J, Zhang J, Chen X (2017). Probiotics for the treatment of atopic dermatitis in children: a systematic review and meta-analysis of randomized controlled trials. Front Cell Infect Microbiol.

[89] Hulshof L, Van't Land B, Sprikkelman AB, Garssen J. Role of microbial modulation in management of atopic dermatitis in children. Nutrients*.* 2017 ;9. pii: E854. 10.3390/nu9080854 PMC5579647 28792444

[90] Zhang GQ, Hu HJ, Liu CY, Zhang Q, Shakya S, Li ZY (2016). Probiotics for prevention of atopy and food hypersensitivity in early childhood: a PRISMA-compliant systematic review and meta-analysis of randomized controlled trials. Medicine (Baltimore).

[91] Zuccotti G, Meneghin F, Aceti A (2015). Italian Society of Neonatology. Probiotics for prevention of atopic diseases in infants: systematic review and meta-analysis. Allergy.

[92] Benninga MA, Faure C, Hyman PE (2016). Childhood functional gastrointestinal disorders: neonate/toddler. Gastroenterology.

[93] Jeffrey S. H, Di Lorenzo C (2016). Functional gastrointestinal disorders: child/ adolescents. Gastroenterology.

[94] Ferreira-Maia AP, Matijasevich A, Wang YP (2016). Epidemiology of functional gastrointestinal disorders in infants and toddlers: a systematic review. World J Gastroenterol.

[95] Boronat AC, Ferreira-Maia AP, Matijasevich A (2017). Epidemiology of functional gastrointestinal disorders in children and adolescents: a systematic review. World J Gastroenterol.

[96] Savino F, Cordisco L, Tarasco V (2010). Lactobacillus reuteri DSM 17938 in infantile colic: a randomized, double-blind, placebo-controlled trial. Pediatrics..

[97] Chau K, Lau E, Greenberg S (2015). Probiotics for infantile colic: a randomized, double-blind, placebo controlled trial investigating Lactobacillus reuteri DSM 17938. J Pediatr.

[98] Sung V, Hiscock H, Tang ML (2014). Treating infant colic with the probiotic Lactobacillus reuteri: double blind, placebo controlled randomised trial. BMJ..

[99] Sung V, D'Amico F, Cabana MD et al. *Lactobacillus reuteri* to treat infant colic: a meta-analysis. Pediatrics. 2018; 141. pii: e20171811. 10.1542/peds.2017-1811 29279326

[100] Szajewska H, Gyrczuk E, Horvath A (2013). Lactobacillus reuteri DSM 17938 for the management of infantile colic in breastfed infants: a randomized, double-blind, placebo-controlled trial. J Pediatr.

[101] Furness JB (2012). The enteric nervous system and neurogastroenterology. Nat Rev Gastroenterol Hepatol.

[102] Verdu EF (2009). Probiotics effects on gastrointestinal function: beyond the gut?. Neurogastroenterol Motil.

[103] Mukhtar K, Nawaz H (2019). Abidet S functional gastrointestinal disorders and gut-brain axis: what does the future hold?. World J Gastroenterol.

[104] Sudo N, Chida Y, Aiba Y (2004). Postnatal microbial colonization programs the hypothalamic-pituitary-adrenal system for stress response in mice. J Physiol.

[105] Harris LA, Baffy N (2017). Modulation of the gut microbiota: a focus on treatments for irritable bowel syndrome. Postgrad Med.

[106] Newlove-Delgado TV, Martin AE, Abbott RA (2017). Dietary intervention for recurrent abdominal pain in children. Cochrane Database Syst Rev.

[107] Bauserman M, Michail S (2005). The use of Lactobacillus GG in irritable bowel syndrome in children: a double blind randomized control trial. J Pediatr.

[108] Francavilla R, Miniello V, Magistà AM (2010). A randomised controlled trial of Lactobacillus GG in children with functional abdominal pain. Pediatrics.

[109] Guandalini S, Magazzù G, Chiaro A (2010). VSL#3 improves symptoms in children with irritable bowel syndrome: a multicenter, randomized, placebo-controlled, double blind, crossover study. J Pediatr Gastroenterol Nutr.

[110] Giannetti E, Maglione M, Alessandrella A (2017). A mixture of 3 bifidobacteria decreases abdominal pain and improves the quality of life in children with irritable bowel syndrome: a multicenter, randomized, double-blind, placebo-controlled, crossover trial. J Clin Gastroenterol.

[111] Ding FLC, Karkhaneh M, Zorzela L (2019). Probiotics for paediatric functional abdominal pain disorders: a rapid review. Paediatr Child Health.

[112] Gawrońska A, Dziechciarz P, Horvath A, Szajewska H (2007). A randomized double-blind placebo-controlled trial of Lactobacillus GG for abdominal pain disorders in children. Aliment Pharmacol Ther.

[113] Kianifar H, Jafari SA, Kiani M (2015). Probiotic for irritable bowel syndrome in pediatric patients: a randomized controlled clinical trial. Electron Physician.

[114] Jadrešin O, Hojsak I, Mišak Z (2017). Lactobacillus reuteri DSM 17938 in the treatment of functional abdominal pain in children. J Pediatr Gastroenterol Nutr.

[115] Maragkoudaki M, Chouliaras G, Orel R (2017). Lactobacillus reuteri DSM 17938 and a placebo both significantly reduced symptoms in children with functional abdominal pain. Acta Paediatr.

[116] Weizman Z, Abu-Abed J, Binsztok M (2016). *Lactobacillus reuteri* DSM 17938 for the management of functional abdominal pain in childhood: a randomized, double-blind, placebo-controlled trial. J Pediatr.

[117] Romano C, Ferrau V, Cavataio F (2014). Lactobacillus reuteri in children with functional abdominal pain. J Paediatr Child Health.

