# Correction to: Probiotics’ efficacy in paediatric diseases: which is the evidence? A critical review on behalf of the Italian Society of Pediatrics

**DOI:** 10.1186/s13052-020-00880-x

**Published:** 2020-08-17

**Authors:** Massimo Martinelli, Giuseppe Banderali, Marisa Bobbio, Elisa Civardi, Alberto Chiara, Sofia D’Elios, Andrea Lo Vecchio, Mattia Olivero, Diego Peroni, Claudio Romano, Mauro Stronati, Renato Turra, Irene Viola, Annamaria Staiano, Alberto Villani

**Affiliations:** 1 Department of Translational Medical Science, Section of Pediatrics, University of Naples “Federico II”, Via S. Pansini, 5, 80131 Naples, Italy; 2 ClinicalDepartment of Pediatrics and Neonatology, San Paolo Hospital, ASST Santi Paolo e Carlo, University of Milan, Milan, Italy; 3 ASL TO4 Piemonte, Turin, Italy; 4 Neonatal Intensive Care Unit, Fondazione IRCCS Policlinico San Matteo Pavia, Pavia, Italy; 5 Department of Clinical and Experimental Medicine, Section of Pediatrics, University of Pisa, Pisa, Italy; 6 Pediatric Gastroenterology and Cystic Fibrosis Unit, University of Messina, Messina, Italy; 7 Pediatric and Infectious Disease Unit, Bambino Gesù Children’s Hospital, IRCCS, Rome, Italy

**Correction to: Ital J Pediatr 46, 104 (2020)**

**https://doi.org/10.1186/s13052-020-00862-z**

One of the studies included in this review [1] assessed a probiotic formulation known as VSL# 3. The probiotic formulation that was assessed in this study is now known by the generic name ‘De Simone Formulation’. The current product known as VSL# 3 is not the same formulation as the original product invented by Professor De Simone. The authors would like to apologise for any inconvenience caused.
